# Treatment Compliance as a Major Barrier to Optimal Cervical Cancer Treatment in Guatemala

**DOI:** 10.1200/JGO.18.00243

**Published:** 2019-05-08

**Authors:** Abigail S. Zamorano, Joaquin Barnoya, Eduardo Gharzouzi, Camaryn Chrisman Robbins, Emperatriz Orozco, Sarita Polo Guerra, David G. Mutch

**Affiliations:** ^1^ Washington University School of Medicine, St Louis, MO; ^2^ Unidad de Cirugía Cardiovascular de Guatemala, Guatemala City, Guatemala; ^3^ Integra Cancer Institute, Guatemala City, Guatemala; ^4^ Instituto de Cancerología, Guatemala City, Guatemala

## Abstract

**PURPOSE:**

Despite being the only hospital to provide comprehensive cervical cancer treatment to many medically underserved Guatemalan women, no assessment of the cervical cancer patient population at the Guatemala Cancer Institute has been performed. To understand the demographics of the patient population, their treatment outcomes, and access to care, we sought to assess treatment compliance of patients with cervical cancer at the Guatemala Cancer Institute and its effects on patient outcomes.

**METHODS:**

A retrospective chart review was conducted of patients with cervical cancer between 2005 and 2007 and assessed for follow-up through December 2015. Demographics and clinical characteristics were tabulated. A Kaplan-Meier curve to model compliance was generated.

**RESULTS:**

Ninety-two patients with invasive cancer were analyzed. Most presented with squamous cell carcinoma (73%) and at locally advanced stages (IIB, 51%; IIIB, 33%). Most (75 of 92, 81.5%) initiated treatment after diagnosis, but 18.5% (17 of 92) were lost to follow-up before treatment initiation. For treatment, 97% received external beam radiation, 84% brachytherapy, and 4% concomitant chemotherapy. Nearly 20% of patients were lost to follow-up in the first 6 months and 65% in the first 5 years. Of the 67 patients who completed treatment, only 15 (16% of the initial cohort) were diagnosed with a recurrence. No deaths were recorded.

**CONCLUSION:**

The low recurrence rate and no documented deaths suggest a correlation with the low compliance rate and poor follow-up. This finding highlights the need to examine more fully the barriers to compliance and access to care among this population to optimize the treatment of cervical cancer.

## INTRODUCTION

Cervical cancer represents a major cause of morbidity and mortality, and the burden of this disease falls especially hard on low- and middle-income countries (LMICs).^1^ In LMICs, cervical cancer is the second most commonly diagnosed cancer and the second leading cause of cancer-related death among women.^2^ Most new cervical cancers (84%) are diagnosed in developing countries, and of the approximately 270,000 deaths each year as a result of cervical cancer, 90% are in LMICs.^3^

**CONTEXT**

**Key Objective**
To deliver optimal care, especially to a medically underserved population with cervical cancer, it is crucial to understand patient demographics and treatment outcomes.
**Knowledge Generated**
We found that many patients do not initiate treatment, and those who do are lost to follow-up soon after treatment. However, to assess the reasons for such differences in the population and make improvements, we found that demographic data are lacking, even at a large academic medical institution.
**Relevance**
Despite being the only hospital to provide comprehensive cervical cancer treatment to many medically underserved Guatemalan women, the Guatemala Cancer Institute has not previously analyzed demographic or treatment information for women with cervical cancer. This is especially important to identify targeted strategies to improve treatment compliance and, therefore, outcomes.

Guatemala, like many Latin American countries, is classified as an LMIC, with 24% of the population earning less than $3.10 per day and 9% earning less than $1.90 per day.^4^ The incidence and mortality of cervical cancer in Guatemala reflects a similar trend as other LMICs. Of all cancers, cervical cancer has the second highest incidence and mortality, with a mortality rate of 12.2 per 100,000 (compared with 2.7 in the United States).^2,5^ This is also meaningful from an economic development standpoint because multiple studies have shown that the death of women, especially young women and mothers, in LMICs places a large economic burden on both the family and the country’s wider economy.^6,7^

As in other LMICs, the low rate of cervical cancer screening in Guatemala is a core contributor to late-stage diagnosis and subsequent mortality. Sixty-two percent of women in 2016 reported never having a Papanicolaou test, and of those, 34% had no knowledge of the test.^8^ Although human papillomavirus vaccines and Pap smears are crucial to cervical cancer prevention, treatment optimization remains imperative because cervical cancer mortality is predicted to increase by 45% by 2030 despite these efforts.^9-11^

Treatment options in Guatemala also are limited. With a population of 15 million, the country has only one hospital that provides comprehensive oncology treatment to the underserved adult population, including surgery, chemotherapy, and radiation treatment: the Guatemala Cancer Institute (INCAN) located in Guatemala City.^11^ The hospital offers colposcopy; ablative and excisional procedures for precancerous lesions; and surgery, radiation treatment, and chemotherapy for invasive disease. However, because INCAN is the only hospital to provide such treatment, many women with cervical cancer have difficulty with accessing quality care immediately upon the onset of symptoms, which results in delayed diagnosis and treatment and increased mortality.

Despite INCAN being the only hospital to provide comprehensive cervical cancer treatment to many medically underserved Guatemalan women, no assessment of the patient population had previously been performed. To understand the demographics of the patient population, their treatment outcomes, and access to care, we sought to assess treatment compliance of patients with cervical cancer at INCAN and its effects on patient outcomes.

## METHODS

We reviewed charts of women diagnosed with cervical cancer at INCAN between 2005 and 2007 and assessed for follow-up until December 2015. Patients were randomly selected from the hospital-based cancer registry: 32 from 2005, 42 from 2006, and 40 from 2007. Of those, 22 patients had cervical intraepithelial neoplasia stage III disease or less and were excluded. There were no additional exclusion criteria. Ninety-two patients with invasive disease were included in the analysis. Data were collected from paper patient charts into a Microsoft Excel (Microsoft Corporation, Redmond, WA) spreadsheet. Demographic and treatment data were then tabulated. Compliance or follow-up was quantified by documentation of hospital visits in the patient’s chart. A Kaplan-Meier curve to model compliance using the known last treatment date to known last follow-up date was created with SAS 9.4 software (SAS Institute, Cary, NC). This study was approved by the institutional review board at INCAN.

## RESULTS

Ninety-two patients were analyzed, and patient demographics are listed in Table 1. Most patients had squamous cell histology (73%) followed by adenocarcinoma (15%). Five patients (5.4%) were diagnosed with stage I disease, 51% (47) with stage II, and 33% (30) with stage III. Only one patient was diagnosed with stage IV disease.

**TABLE 1:**
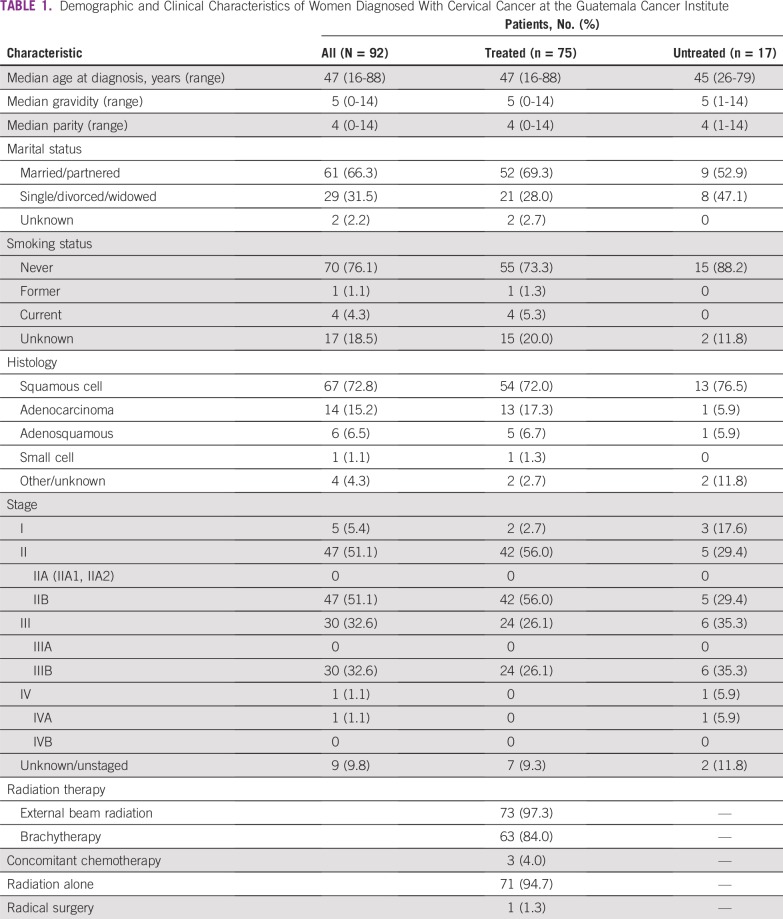
Demographic and Clinical Characteristics of Women Diagnosed With Cervical Cancer at the Guatemala Cancer Institute

Of 92 patients, 17 (18.5%) did not follow up after diagnosis, which left 75 patients (81.5%) who initiated treatment. Of those who initiated treatment, 67 (73%) completed treatment; 60 (65%) were subsequently seen in follow-up at least once. There were no significant differences in age, histology, and stage of disease at the time of diagnosis between those who received any treatment and those who did not (Table 1). With regard to treatment, 73 (97%) of the 75 patients who initiated treatment received external beam radiation, and 63 (84%) received brachytherapy. INCAN did not have a linear accelerator until 2014, so within the time frame that our sample was collected, all patients received cobalt radiation. Only three patients (4%) received concomitant chemotherapy.

**FIG 1:**
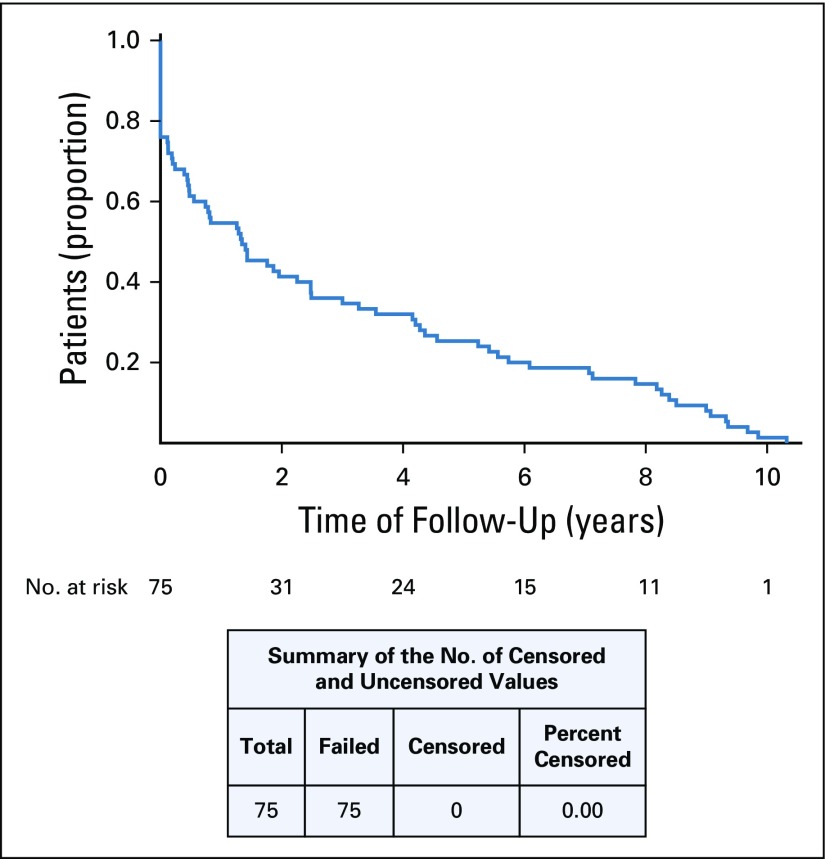
Decline in follow-up after treatment.

As shown in Figure 1, compliance, marked by clinical follow-up, declined appreciably in the first 6 months after treatment conclusion. Eleven (18.3%) of the 60 patients who completed treatment had their final encounter at INCAN within 6 months of treatment completion. At 5 years, 65% of those who completed treatment had been lost to follow-up. Median follow-up was 30 months (range, 1 to 126 months), with an interquartile range of 67. One patient remained at the end of the follow-up period. Fifteen patients (16.3%) were diagnosed with a recurrence. We found no evidence of hospital deaths or deaths after treatment completion.

## DISCUSSION

Several aspects contribute to the high burden of cervical cancer in Guatemala, as demonstrated by this study. First, most women are diagnosed with locally advanced disease that likely correlates with the onset of symptoms. Second, nearly one quarter of women do not initiate any treatment after diagnosis, and the majority of those who receive treatment do not receive the standard of care in the form of radiation with concomitant chemotherapy. Finally, low compliance rates, as highlighted in our study, complicate the ability to optimize cervical cancer treatment.

In terms of treatment regimens, the fact that only three patients received concomitant chemotherapy with radiation illustrates the challenges of providing the standard of care to an under-resourced population. Although the limitations that most LMICs face in providing adequate radiation therapy have been well documented,^12-14^ those related to chemotherapy delivery remain largely unexplored. A 2015 review of cervical cancer in LMICs in Africa noted that less than 5% of those needing chemotherapy received it, that appropriate chemotherapy was grossly unavailable, and that the average price of medications was equal to 7 months of income.^15^ At INCAN, discounts are offered for some limited treatment, but no assistance is provided for chemotherapy, which makes its use prohibitive for many patients.^16^ Although the reasons for not administering concomitant platinum therapy were not documented in the patient charts, we suppose a leading barrier is economic concerns. This, however, requires additional research to confirm and rule out other obstructions, such as the distance to the treatment center and the ability to find and pay for lodging in Guatemala City while receiving treatment.

With an understanding of the poor rate of follow-up after treatment, it should be of no surprise that only 16% of patients were diagnosed with a recurrence, not because patients did not experience a recurrence but because they did not present to INCAN when they did. With radiation alone, we would expect a recurrence rate to be 20% to 30% in stage II disease and close to 40% in stage III disease.^17^ Because INCAN is the only place that many indigent patients can receive treatment in Guatemala, those who were lost to follow-up are presumed to have followed the natural course of cervical cancer and died.

We found no hospital deaths or evidence of death in the charts. However, in Guatemala, deaths usually occur at home instead of in the hospital. Although death certificates are created, they are not linked to the government death registry or to patient charts. Assessment of survival among our sample, therefore, was impossible unless the death occurred while in the hospital. Additional practical barriers keep people at home when they are in pain, bedridden, or at the time of death. INCAN has a palliative care service, but transportation, finances, and limited hospital hours and staff all contribute to restricting patient access.^16^

Although compliance during treatment has been examined in an American population,^18^ to our knowledge, our study is the first to demonstrate the dramatic decline in patient compliance before, during, and after treatment in an LMIC. This study was intended to be a pilot review of patients with cervical cancer in Guatemala to provide baseline data on cancer treatment in this population. Although the initial intention was to examine survival rates, the lack of follow-up data made this impossible. The scarcity of organized data or records of patient outcomes thwarts clinical research and is thus a critical barrier to health care improvement in LMICs, especially in the most underserved populations.

This study was not powered to detect statistically significant differences between patient groups. In addition, no interviews were conducted to assess reasons for noncompliance. The study also is subject to the same limitations as that inherent in single-institution, retrospective studies in that these findings may not be generalizable to other hospital systems, even in other under-resourced areas. However, the lessons learned from the barriers to conducting research in and providing care to LMICs as well as the improvements that are necessary to adequately treat marginalized populations can and should be considered by the wider academic world.

Additional data are needed to confirm our findings and to identify the most at-risk demographics and clinical characteristics that determine compliance. The next steps are to look at more-specific aspects of patient demographics to develop targeted strategies to reach those most at risk for compliance with treatment and follow-up. We hope that these data illuminate the need for targeted strategies to improve treatment compliance and, therefore, outcomes.
